# CX3CL1–CX3CR1 axis promotes restraint stress-induced thymic involution through macrophage-mediated thymocyte apoptosis

**DOI:** 10.3389/fimmu.2026.1930342

**Published:** 2026-09-15

**Authors:** Yuko Ishida, Yumi Kuninaka, Mizuho Nosaka, Akiko Ishigami, Naofumi Mukaida, Toshikazu Kondo

**Affiliations:** Department of Forensic Medicine, Wakayama Medical University, Wakayama, Japan

**Keywords:** CX3CL1, CX3CR1, macrophage, restraint stress, thymic involution, thymocyte apoptosis

## Abstract

**Background:**

Stress-induced thymic involution is characterized by rapid thymocyte loss and is largely attributed to glucocorticoid signaling. However, the local immune mechanisms involved remain unclear. We investigated the role of the CX3CL1–CX3CR1 axis in restraint stress (RS)-induced thymic involution.

**Methods:**

Wild-type (WT) and *Cx3cr1*-deficient mice were subjected to repeated RS. Thymic morphology, CX3CL1/CX3CR1 expression, apoptosis-related pathways, and stress-related hormonal responses were examined. The effects of CX3CL1 on corticosterone (CORT)-induced thymocyte apoptosis *in vitro* and exogenous CORT administration *in vivo* were also evaluated.

**Results:**

RS increased CX3CL1 and CX3CR1 expression in thymic F4/80-positive macrophages. *Cx3cr1* deficiency markedly attenuated RS-induced thymic involution, preserved thymic architecture, and reduced thymocyte apoptosis. RS-induced Fas ligand expression in thymic macrophages and caspase-3 activation in the thymic cortex were also attenuated by *Cx3cr1* deficiency. CX3CL1 did not enhance CORT-induced thymocyte apoptosis *in vitro*, and a single CORT administration did not reproduce the marked thymic involution induced by repeated RS. CX3CR1 expression was also detected in hypothalamic microglia.

**Conclusion:**

CX3CL1–CX3CR1 signaling contributes to RS-induced thymic involution through CX3CR1-dependent mechanisms involving macrophage-associated FasL-mediated thymocyte apoptosis and modulation of the corticosterone response, suggesting coordinated local immune and neuroendocrine mechanisms underlying stress-induced thymic involution.

## Introduction

Stress-induced thymic involution is an acute and reversible process characterized by a rapid reduction in thymic size, mainly due to the depletion of CD4^+^CD8^+^ double-positive thymocytes and subsequent impairment of naïve T cell output (1, 2). Unlike age-associated thymic involution, acute thymic atrophy occurs in response to various physiological and pathological stressors, including infection, malnutrition, and systemic inflammation (1, 3, 4). Activation of the hypothalamic–pituitary–adrenal (HPA) axis and subsequent glucocorticoid release are recognized as major mechanisms driving stress-induced thymocyte apoptosis; however, the local immune mechanisms regulating thymic damage remain incompletely understood.

Accumulating evidence indicates that immune cells and inflammatory mediators within the thymic microenvironment contribute to stress-induced thymic involution. Several cytokines, including leukemia inhibitory factor (LIF), have been reported to promote thymic atrophy through both systemic endocrine effects and local inflammatory responses (5, 6). Moreover, interactions between the immune and neuroendocrine systems play essential roles in regulating stress responses, suggesting that immune mediators may serve as important links between psychological stress and thymic dysfunction (7, 8).

Chemokines are critical regulators of immune cell migration and inflammatory responses. CX3CL1 (fractalkine) is a unique CX3C chemokine that acts through its specific receptor CX3CR1, which is predominantly expressed on monocytes, macrophages, and microglia (9–11). The CX3CL1–CX3CR1 axis regulates macrophage recruitment, activation, and tissue remodeling in various inflammatory diseases (12–14). Recent studies have further demonstrated that CX3CR1-expressing macrophages contribute to tissue homeostasis and inflammatory responses and that CX3CL1–CX3CR1 signaling can regulate macrophage migration and functional phenotype under pathological conditions (15, 16). In addition, CX3CR1-mediated interactions between neurons and microglia contribute to neuroimmune communication and influence stress-related responses in the central nervous system (17–19). Recent evidence further suggests that dynamic regulation of CX3CR1 may contribute to microglial adaptation and resilience to stress-related neuronal disturbances (20).

Despite the established roles of CX3CL1–CX3CR1 signaling in inflammation and neuroimmune regulation, its contribution to acute stress-induced thymic involution remains unknown. Given the involvement of macrophages and HPA axis regulation in stress responses, we hypothesized that CX3CL1–CX3CR1 signaling promotes thymic atrophy through macrophage-mediated thymocyte apoptosis and neuroendocrine modulation. In this study, using a restraint stress model and *Cx3cr1*-deficient mice, we investigated the functional role and underlying mechanisms of the CX3CL1–CX3CR1 axis in stress-induced thymic involution.

## Methods

### Animals

Pathogen-free 8-week-old male C57BL/6 mice were obtained from Sankyo Laboratories (Tokyo, Japan) and designated as wild-type (WT) mice. *CX3CR1-deficient* (*Cx3cr1*^-/-^) mice with the C57BL/6 genetic background were a generous gift from Drs. P. M. Murphy and J. L. Gao (National Institute of Allergy and Infectious Diseases, National Institutes of Health, Bethesda, MD) (13). All animals were housed individually in cages under specific pathogen-free conditions during the experiments. Age- and sex-matched mice were used for the experiments. Animals were maintained at 21 ± 2°C under a 12-h light/12-h dark cycle with free access to standard chow and water (*ad libitum*). Individual housing was initiated 1 week before the start of the experiments and maintained throughout the study. Control and experimental groups were handled under identical environmental and experimental conditions. All animal experiments complied with the standards set by the Guidelines for the Care and Use of Laboratory Animals at the Wakayama Medical University (Approved No. 1279; September 4, 2025).

### Restraint stress

WT and *Cx3cr1^−/−^* mice were subjected to restraint stress as previously described, with minor modifications (2). Mice were immobilized individually in well-ventilated cylindrical wire mesh restrainers sized 12 cm (length) × 3 cm (diameter) that were closed off at both ends at 9:00 AM. The restrained mice were maintained horizontally in their home cages during the restraint sessions and released into the same cages during the free sessions. They were restrained for 3 h starting at 9:00 AM daily followed by a 21 h recovery period. Mice were restrained for three consecutive days. Food and water were provided during the recovery period ad libitum; therefore, food and water deprivation during the restraint sessions should be considered as part of the stress. Control animals were housed in their home cages with food and water provided ad libitum at all times. In some experiments, WT mice were injected intraperitoneally with anti-mouse CX3CL1 (100 μg/200 μl/mouse) or control-immunoglobulin G (IgG) as a control on days −1, 0, 1, and 2 relative to the initiation of restraint stress. At the end of the final restraint stress session, animals were euthanized by CO_2_ inhalation at 12:00 PM. CO_2_ was introduced gradually into a 20-L euthanasia chamber at a flow rate of 6 L/min, corresponding to 30% of the chamber volume per minute, until respiration ceased. Death was confirmed before sample collection. Blood or thymic tissues were then collected, respectively. Blood was drawn immediately after euthanasia by cardiac puncture. Plasma samples were collected in 50 mM EDTA in phosphate-buffered saline (PBS) and stored at -20°C. The thymi were excised and put into 1 ml of cell culture medium. The weight of each organ was recorded. Six mice were used in each treatment group whenever possible or the data from parallel experiments were combined for the statistical analysis.

### Corticosterone administration

WT and *Cx3cr1^−/−^* mice were administered a single intraperitoneal injection of CORT (4 mg/mouse; 27840, Sigma, St. Louis, MO) dissolved in 100 μL olive oil (21). Control mice received 100 μL olive oil alone. Twenty-four hours after administration, mice were euthanized by CO_2_ inhalation using the same procedure described above, and the thymi were excised and weighed. The thymus weight/body weight ratio (mg/g) was calculated as an index of thymic atrophy. Four mice were used in each group.

### Measurement of corticosterone (CORT) and adrenocorticotropic hormone in plasma

Blood samples were collected at the end of the final stress session (12:00 PM). Plasma CORT concentrations were measured with a CORT ELISA kit from IBL international (#RE52211, Hamburg, Germany) according to the manufacturer’s instructions. The levels of ACTH were measured using an ACTH ELISA kit from MD bioproducts (#M046006, Oakdale, MN) according to the manufacturer’s instructions.

### Histopathological and immunohistochemical analyses

Histopathological and immunohistochemical analyses were performed as previously described, with minor modifications (13). Thymic tissues were fixed in 10% neutral-buffered formalin and then embedded in paraffin to prepare sections. The deparaffinized sections were subjected to hematoxylin and eosin (H&E) staining for histopathological analysis. Immunohistochemical analysis was performed. Deparaffinized thymic sections of mice were immersed in 0.3% H_2_O_2_ in methanol for 30 min to eliminate endogenous peroxidase activities. The sections were further incubated with PBS containing 1% normal serum corresponding to the secondary antibody (Ab) and 1% bovine serum albumin to reduce nonspecific reactions. Antigen retrieval was performed by boiling in commercialized retrieval buffer (901-CB911-071117, Biocare Medical, Pacheco, CA and S1699, DAKO, Glostrup, Denmark). Subsequently, sections were incubated overnight at 4°C with the following primary antibodies (Abs): goat anti-mouse CX3CL1 polyclonal antibody (pAb) (1:200, sc-7227, SantaCruz, Dallas, TX) or rabbit anti-mouse CX3CR1 pAb (1:100, PAB16479, Abnova, Taipei city, Taiwan). After incubation with horseradish peroxidase (HRP)-conjugated rabbit anti-goat or goat anti-rabbit IgG, immune complexes were visualized using Tyramide Amplification Buffer Plus (22029, Biotium, Fremont, CA) with Tyramide Signal Amplification Kits (33020, Biotin-XX, Biotium) and LSAB2 Streptavidin-HRP (K1016, Dako, Carpinteria, CA) according to the manufacturer’s instructions. Histopathological and immunohistochemical findings were evaluated without any prior knowledge of the experimental procedures.

### Double-color immunofluorescence analysis

Double-color immunofluorescence analysis was performed to identify CX3CL1- and CX3CR1-expressing cells, cortical and medullary cells, and apoptotic cells in the thymus. Briefly, deparaffinized sections were incubated with PBS containing 1% normal donkey serum and 1% bovine serum albumin (BSA) to reduce non-specific reactions. In double-color analysis, the sections were further incubated in combinations of rat anti-mouse F4/80 monoclonal antibody (mAb) (clone BM8, T-2006, BMA Biomedicals, Switzerland) and anti-CX3CL1 or anti-CX3CR1, rabbit anti-mouse keratin 5 pAb (1:100, GTX113219, GeneTex, Irvine, CA) and guinea pig anti-mouse keratin 8 pAbs (1:100, #GP-K8, Progen, Heidelberg, GmbH), or rabbit anti-mouse caspase-3 mAb (1:100, clone Asp175, 5A1E, #9664, Cell Signaling, Danvers, MA), or rat anti-mouse Iba1 mAb (1:100, clone HL22-RT, GTX635400, GeneTex, Irvine, CA) and anti-CX3CR1. The sections were incubated for 30 min at room temperature with the appropriate combination of the following fluorochrome-conjugated secondary antibodies: Cy3-conjugated donkey anti-goat and anti-rabbit IgG pAbs (1:400, 711-165-152), and Alexa Fluor 488-conjugated donkey anti-rat and anti-guinea pig IgG pAbs (1:50, 706-545-148) obtained from Jackson Immunoresearch Laboratories (West Grove, PA). Images were observed with a fluorescence microscope (BZ-X710 All-in-One Fluorescence Microscope, KEYENCE, Itasca, IL). Notably, F4/80 was used to identify macrophages in the thymus, as it is an established marker for mouse macrophages. In contrast, we used Iba1—a widely recognized marker for resident microglia in the central nervous system—exclusively for the analysis of the hypothalamic region surrounding the third ventricle. Since F4/80 expression in microglia is relatively weak and variable, Iba1 allows for more reliable identification of hypothalamic microglia.

### Terminal deoxynucleotidyl transferase dUTP nick-end labeling staining

Apoptotic cells in thymic tissues were detected by TUNEL staining as previously described (22), with minor modifications, using an *In Situ* Cell Death Detection Kit, POD (#1684817; Merck, Darmstadt, Germany). The deparaffinized thymic sections were first incubated with Proteinase K working solution (provided in the kit) for 30 min at 37°C. After incubation with the TUNEL reaction mixture (provided in the kit) for 60 min at 37°C, the tissue sections were further incubated with POD converter (provided in the kit) for 30 min at 37°C. Then the tissue sections were incubated with 3, 3′-diaminobenzidine (DAB) substrate for 10 min at room temperature. Finally, the tissue sections were observed and documented using a light microscope.

### Quantitative image analysis

Quantitative image analysis was performed using Adobe Photoshop 2026 (Adobe Systems, San Jose, CA, USA). Prior to analysis, the thymic tissue region was manually delineated in each image, and areas outside the tissue were excluded from the analysis. For bright-field immunohistochemical images, the immunoreactive chromogen signal was separated from the counterstain using color deconvolution. The area occupied by the extracted immunopositive signal was measured and normalized to the total tissue area, and the results were expressed as the percentage of immunopositive area. For double-color immunofluorescence images, images were separated into red, green, and blue channels, and the area occupied by the red or green fluorescence signal corresponding to each marker was measured. Fluorescence-positive areas were normalized to the total tissue area and expressed as percentages. Identical image-processing and thresholding parameters were applied to all samples within the same staining experiment.

### Real-time quantitative polymerase chain reaction

Real-time quantitative PCR was performed as previously described (13), with minor modifications. Total RNAs were extracted from mouse thymic tissues using ISOGEN (Nippon Gene, Toyama, Japan), according to the manufacturer’s instructions. Total RNAs were reverse transcribed to complementary DNA (cDNA) using Prime Script Reverse Transcriptase (Takara Bio, Shiga, Japan) with oligo(dT)_15_ primers. PCR was carried out using SYBR Premix Ex Taq II (Takara Bio) with specific primer sets (Takara Bio, **Table 1**). Amplification and detection of messenger RNA (mRNA) were performed using Thermal Cycler Dice Real Time System (TP800, Takara Bio) according to the manufacturer’s instructions. To normalize mRNA levels, transcript levels of *Actb* were determined in parallel for each sample, and relative transcript levels were corrected by normalization based on *Actb* transcript levels.

**Table 1:** Sequences of primers used for real-time RT-PCR.

Transcript	Sequence
*Cx3cr1*	(F) 5’-CCGGTCTCATTTGCAGGCTTA-3’ (R) 5’-TGGGAATTGAACTTGGGACCTC-3’
*Cx3cl1*	(F) 5’-ACCTATGGCCCTGACATCATCAC-3’ (R) 5’-CTTGCCAGCCCTCAGAATCAC-3’
*Fas*	(F) 5’-GAAGATGCACACTCTGCGATGAA-3’ (R) 5’-TCGCAGTAGAAGTCTGGTTTGCAC-3’
*Fasl*	(F) 5’-GGAACTGGCAGAACTCCGTGA-3’ (R) 5’-AGATCAGAGCGGTTCCATATGTGTC-3’
*Actb*	(F) 5’-CATCCGTAAAGACCTCTATGCCAAC-3’ (R) 5’-ATGGAGCCACCGATCCACA-3’

(F), Forward primer; (R), Reverse primer.

### Annexin V apoptosis assay *in vitro*

The sensitivity of thymocytes to glucocorticoid-induced apoptosis was evaluated using Annexin V/propidium iodide (PI) staining, with some modifications to a previously reported method (23). Thymocytes were prepared from mouse thymic tissues by passing the tissues through a sterile nylon mesh, 400 μm, followed by centrifugation at 800 × g for 5 min at 4°C, and were then seeded onto 12-well plates at a density of 2 × 10^7^ cells/well with RPMI1640 supplemented with 10% fetal bovine serum (FBS). Subsequently, thymocytes were treated with corticosterone (300 ng/ml, 27840, Sigma, St. Louis, MO) and recombinant mouse CX3CL1 (0.1, 1, or 10 ng/ml) for 16 h. The apoptotic effect of CX3CL1 on thymocytes was analyzed using Annexin V-fluorescein isothiocyanate (FITC) Kit (MEBCYTO Apoptosis Kit, MBL, 4700, Nagoya, Japan). Briefly, cells were collected and resuspended in 85 μl Binding Buffer. Subsequently, 10 μl annexin V-FITC and 5 μl propidium iodide were added to the cell suspension and incubated at room temperature for 15 min in the dark. After incubation, 400 μl Binding Buffer was added, and cell samples were measured by Flow Cytometry (CytoFLEX S, Beckman Coulter, Brea, CA) and FlowJo software v10.5 (Becton, Dickinson and Company, Franklin Lakes, NJ).

### Statistical analysis

Data were expressed as the mean ± standard error of the mean (SEM). For the comparison between WT and *Cx3cr1*^-/-^ mice at multiple time points, 2-way analysis of variance (ANOVA) followed by Tukey's *post-hoc* test was used. To compare the values between two groups, a two-sided unpaired Student’s *t* test was performed. *P* < 0.05 was considered statistically significant. All statistical analyses were performed using Statcel3 software under the supervision of a medical statistician.

### Study approval

All animal experiments were approved by the Ethics Committee of Animal Research, Wakayama Medical University (Approved No. 1279; September 4, 2025). All methods were performed in accordance with the relevant guidelines and regulations.

### Data availability

The raw data supporting the conclusions of this article will be made available by the corresponding author (Toshikazu Kondo), without undue reservation.

## Results

### Enhanced expression of *Cx3cl1* and *Cx3cr1* in thymi after RS

RS application significantly enhanced *Cx3cl1* and *Cx3cr1* mRNA expression in the thymi (**Figures 1a, b**). There was no significant enhancement after RS of other chemokines such as *Ccl2, Ccl3, Ccl4*, and *Ccl5*, their receptors *Ccr2* and *Ccr5*, or inflammatory cytokines such as *Il1b, Il6, Il12p35*, and *Il12p40* compared with controls (**Supplementary Figure 1**). Immunohistochemical analysis revealed few CX3CL1- and CX3CR1-positive cells in the thymi of control mice, whereas the expression of both CX3CL1 and CX3CR1 was markedly increased after RS (**Figures 1c, e**). Quantitative image analysis confirmed that the CX3CL1- and CX3CR1-positive areas were significantly increased after RS compared with those in control mice (**Figures 1d, f**). Double-color immunofluorescence analysis further demonstrated that CX3CL1 and CX3CR1 were predominantly expressed in F4/80-positive macrophages in the thymus (**Figures 1g, h**). These observations indicated the potential involvement of the CX3CL1–CX3CR1 axis in RS-induced thymic atrophy.

**Figure 1:**
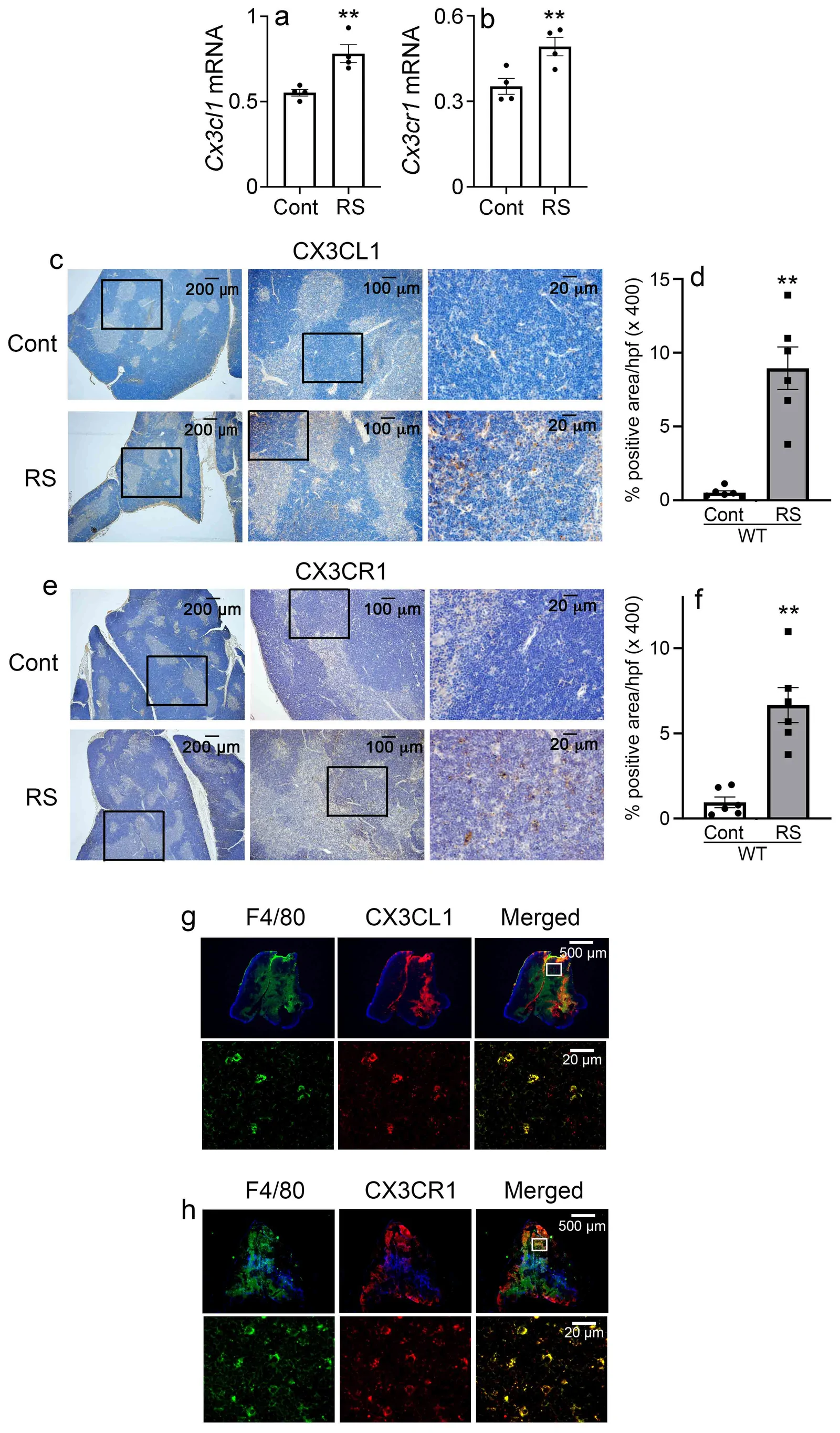
The expression of *Cx3cl1* and *Cx3cr1* in thymi after restraint stress (RS). Real-time RT-PCR analyses for the gene expression of *Cx3cl1* **(a)** and *Cx3cr1* **(b)** in thymi (n = 4 mice). All values represent mean ± standard error of the mean (SEM). **P < 0.01 vs. controls. Immunohistochemical analyses using anti-CX3CL1 **(c, d)** and anti-CX3CR1 **(e, f)** in thymi. Representative results from six individual animals are shown. Frames in the left columns were displayed at a higher magnification in the right columns. Cell type expressing CX3CL1 **(g)** and CX3CR1 **(h)** in thymi after RS. Representative results from 6 individual animals are shown. Signals were merged digitally. Frames in the upper columns were displayed at a higher magnification in the lower columns.

### The involvement of the CX3CL1–CX3CR1 axis in RS-induced thymic atrophy

To address the role of the CX3CL1–CX3CR1 axis in RS-induced thymic atrophy, WT and *Cx3cr1^−/−^* mice were subjected to RS. There were no apparent differences in thymic size between control WT and *Cx3cr1^−/−^* mice (**Figure 2a**). As shown in **Figure 2b**, RS caused an approximately 40% reduction in thymus weight in WT mice but only about a 10% reduction in *Cx3cr1^−/−^* mice. Fluorescence microscopy of thymic tissue sections stained with anti-keratin 5 (K5; medullary thymic epithelial cells) and anti-keratin 8 (K8; cortical thymic epithelial cells) antibodies revealed a marked reduction in the cortical region in WT mice after RS, whereas this alteration was attenuated in *Cx3cr1^−/−^* mice (**Figure 2c**). Quantitative image analysis demonstrated that the K8/K5-positive area ratio was significantly increased after RS in WT mice, whereas this increase was significantly attenuated in *Cx3cr1^−/−^* mice (**Figure 2d**). The distortion of thymic architecture after RS and its attenuation in *Cx3cr1^−/−^* mice were also confirmed by H&E staining (**Figure 2e**). These results indicated that the CX3CL1–CX3CR1 axis plays an important role in mediating stress-induced thymic involution.

**Figure 2:**
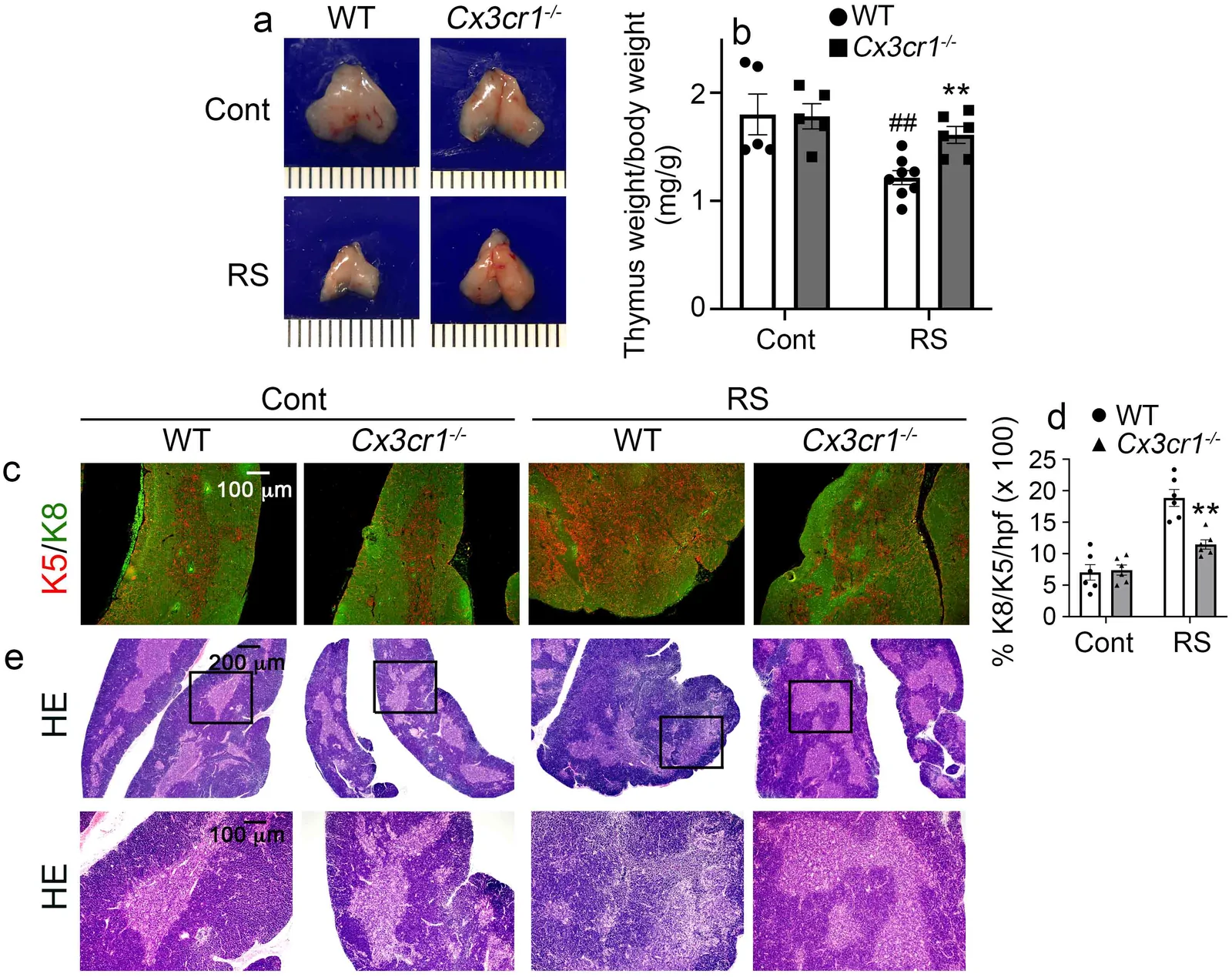
RS-induced thymic atrophy under conditions of disruption of the CX3CL1-CX3CR1 axis. **(a)** Macroscopic thymic atrophy in WT and *Cx3cr1^-/-^* mice. **(b)** Thymus weight/body weight (mg/g) (n = 5 to 8 mice). All values represent mean ± SEM. **P < 0.01 vs. WT-RS; ^##^*P* < 0.01 vs. WT-control. **(c)** Fluorescence microscopy on thymic tissue sections stained with anti-keratin 5 (medullary thymic epithelial cells) and anti-keratin 8 (cortical thymic epithelial cells) antibodies. **(d)** Quantitative image analysis of K8/K5 was performed, and the results were expressed as a percentage. All values represent mean ± SEM (n=6 mice). **P < 0.01 vs. WT-RS. **(e)** Histopathological changes in the thymus of WT and *Cx3cr1*^-/-^ mice by H&E staining. Representative results from 6 individual animals are shown. Frames in the upper columns were displayed at a higher magnification in the lower columns.

### Stress-induced changes in hypothalamus-pituitary-adrenal axis hormones are affected by deficiency of CX3CR1

Corticosteroids exert negative feedback regulation on stress hormones in the HPA axis by suppressing the secretion of ACTH and corticotropin-releasing hormone (24). The levels of CORT and ACTH in blood samples harvested after the termination of RS were tested with commercial ELISA kits. The results showed that there was no significant difference in both CORT and ACTH levels between unstressed WT and *Cx3cr1^-/-^* mice (**Figures 3a, b**). RS significantly elevated CORT levels in WT but not in *Cx3cr1^-/-^* mice (**Figure 3a**). On the other hand, the level of ACTH was significantly reduced in RS-treated WT mice, whereas there was no remarkable change in ACTH levels in RS-treated *Cx3cr1^-/-^* mice (**Figure 3b**). These results suggested that ACTH secretion was suppressed by the elevated CORT levels in RS-treated WT mice. Collectively, these findings indicate that CX3CR1 deficiency alters the CORT and ACTH responses to restraint stress, suggesting the involvement of CX3CR1 signaling in the neuroendocrine response to repeated restraint stress.

**Figure 3:**
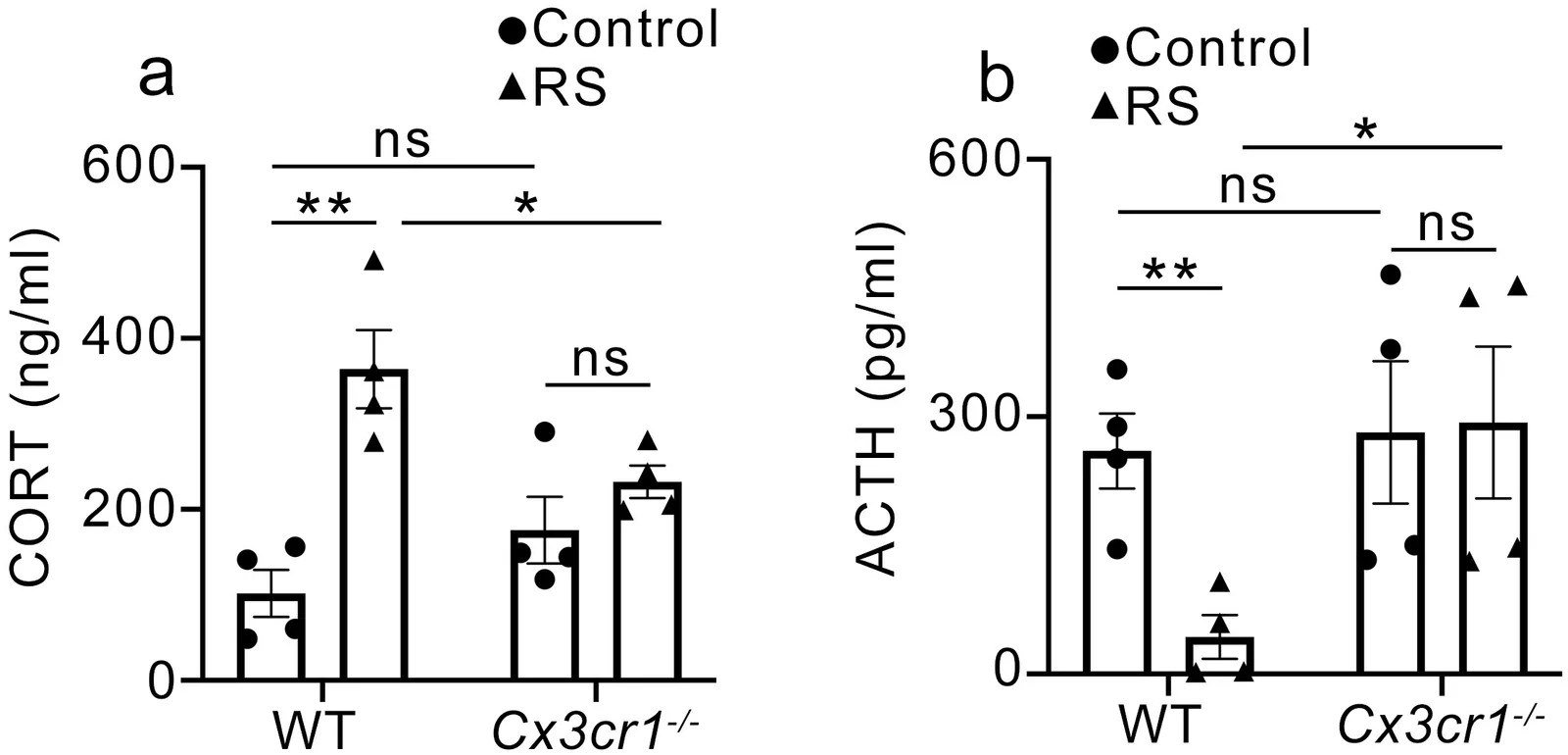
Changes in hormone levels in response to RS. **(a)** CORT levels in plasma of RS-treated mice. **(b)** ACTH levels in plasma of RS-treated mice. All values represent mean ± SEM. Statistical significance was analyzed using two-way ANOVA followed by Tukey’s multiple comparisons test. **P < 0.01; *P < 0.05; ns, not significant (n = 4 mice).

### Effect of CX3CL1 on CORT-induced thymocyte apoptosis

To evaluate the role of CX3CL1 in thymocytes, we then examined thymocytes treated with CORT *in vitro*. CORT treatment increased the proportion of Annexin V-positive apoptotic cells, but there was no change in apoptotic cells at any dose of recombinant CX3CL1 added at the same time (**Figure 4**). These observations indicated that CX3CL1 had little effect on thymocyte apoptosis. In fact, neutralization of CX3CL1 in RS-induced thymic atrophy *in vivo* showed that RS caused a similar reduction in thymic weight in both the control and anti-CX3CL1 groups in WT mice (**Supplementary Figure 2**). To further determine whether exogenous CORT could reproduce the thymic changes observed after RS *in vivo*, WT and *Cx3cr1^−/−^* mice were administered a single intraperitoneal injection of CORT (4 mg/mouse), and thymic weight was assessed 24 h later. CORT administration tended to decrease the thymus weight/body weight ratio compared with vehicle treatment in both WT and *Cx3cr1^−/−^* mice; however, the differences did not reach statistical significance (**Supplementary Figure 3**). Moreover, no significant difference was observed between WT and *Cx3cr1^−/−^* mice following CORT administration. These findings suggest that a single administration of CORT under the present experimental conditions was not sufficient to reproduce the marked thymic involution observed after repeated RS.

**Figure 4:**
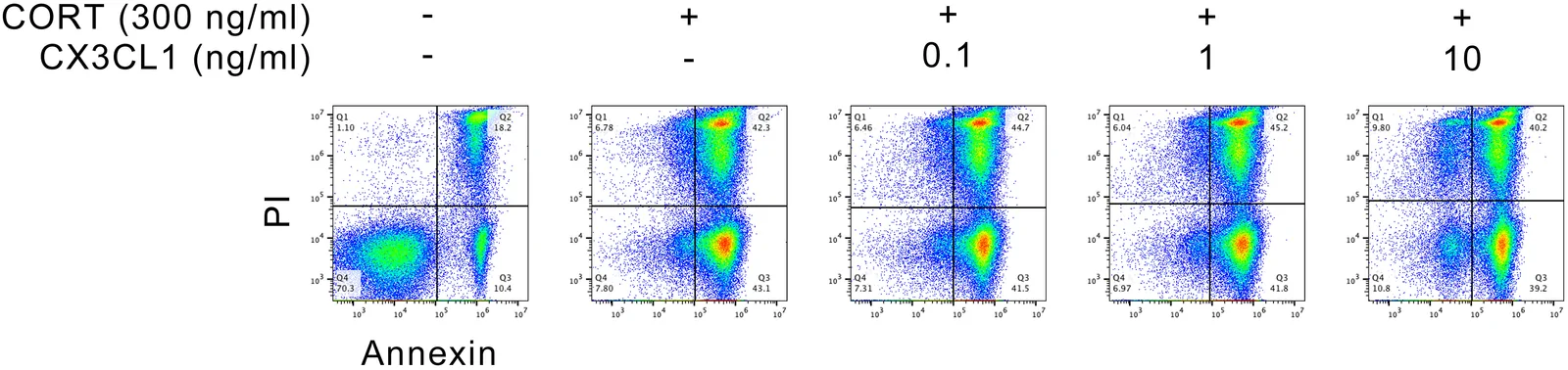
Determination of susceptibilities of thymocytes from WT mice to apoptotic stimuli. Representative results from four individual experiments are shown.

### Apoptotic cells were increased in the thymic cortex after RS

We examined the gene expression of *Fas* and *Fasl* in the thymi. There was no significant difference in the mRNA expression of *Fas* or *Fasl* between WT and *Cx3cr1^−/−^* mice before RS application (**Figures 5a, b**). *Fas* gene expression was not significantly altered by RS in either WT or *Cx3cr1^−/−^* mice (**Figure 5a**). In contrast, RS enhanced *Fasl* expression in the thymi of WT mice, whereas *Cx3cr1^−/−^* mice exhibited significantly lower *Fasl* mRNA expression than WT mice after RS (**Figure 5b**). Double-color immunofluorescence analysis demonstrated that FasL was expressed in F4/80-positive macrophages in the thymus (**Figure 5c**). TUNEL staining revealed few apoptotic cells in the thymi of both WT and *Cx3cr1^−/−^* mice before RS, whereas numerous TUNEL-positive cells were observed in WT mice after RS (**Figure 5d**). Quantitative image analysis confirmed that the TUNEL-positive area was markedly increased after RS in WT mice, whereas this increase was significantly attenuated in *Cx3cr1^−/−^* mice (**Figure 5e**). Immunofluorescence analysis further demonstrated increased caspase-3-positive staining predominantly in the K8-positive cortical region after RS (**Figure 5f**). Quantitative analysis confirmed a marked increase in the caspase-3-positive area after RS compared with that in control WT mice (**Figure 5g**). These findings suggested that CX3CR1 deficiency suppressed RS-induced FasL expression and subsequent thymocyte apoptosis, particularly in the thymic cortex.

**Figure 5:**
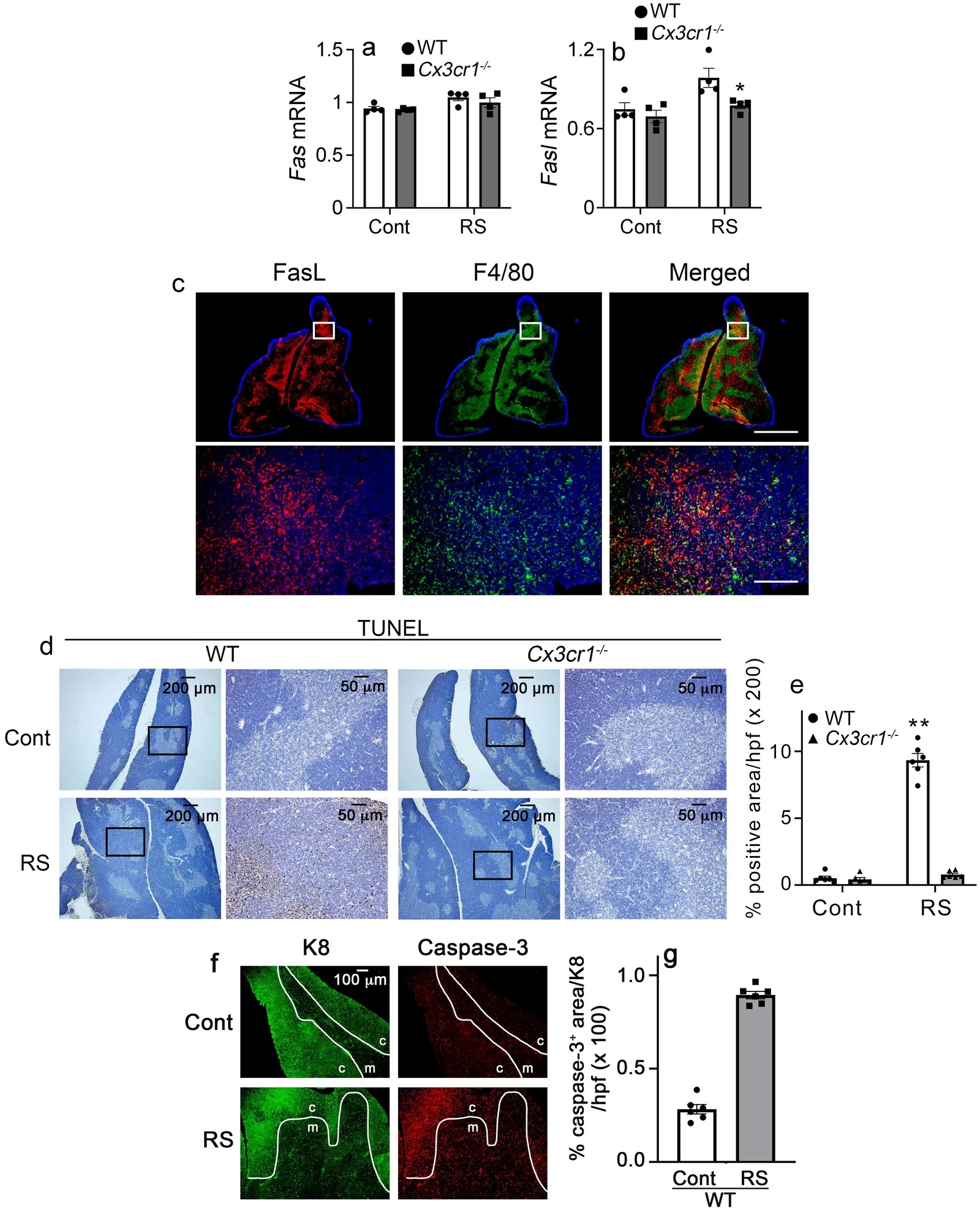
Determination of apoptotic thymocytes in WT and *Cx3cr1^-/-^* mice. Real-time RT-PCR analyses for the gene expression of *Fas* **(a)** and *Fasl* **(b)** in thymi (n = 4 mice). All values represent mean ± SEM. *P < 0.05 vs. controls. **(c)** Double-stained immunofluorescence analysis using anti-FasL antibody and anti-F4/80 antibody. Nuclei were stained with 4′, 6-diamidino-2-phenylindole (DAPI). Frames in the upper columns (Scale bars, 500 μm) were displayed at a higher magnification in the lower columns (Scale bars, 20 μm). Representative results from 4 individual animals are shown. **(d)** TUNEL-positive cells (in brown) in thymi from WT and *Cx3cr1^-/-^* mice. Nuclei were stained with hematoxylin. Representative results from 6 individual animals are shown. **(e)** Quantitative image analysis of TUNEL^+^ areas was performed, and the results were expressed as a percentage. **(f)** Caspase-3 positive cells in thymi before and after RS in WT mice. c, cortical cells (Keratin 8-positive cells); m, medullary cells. Representative results from 6 individual animals are shown. Signals were merged digitally. **(g)** Quantitative image analysis of caspase-3^+^ areas was performed, and the results were expressed as a percentage.

### Microglia expressed CX3CR1 in the hypothalamus after RS

Double-color immunofluorescence analysis showed that some Iba1-positive microglia in the paraventricular nucleus (PVN) of the hypothalamus expressed CX3CR1 both before and after RS (**Figure 6**). MAP2-positive neuronal cells were also positive for CX3CR1 in both control and RS treated WT mice equivalently. These findings suggested a potential involvement of CX3CR1-expressing hypothalamic cells in the neuroendocrine response to RS.

**Figure 6:**
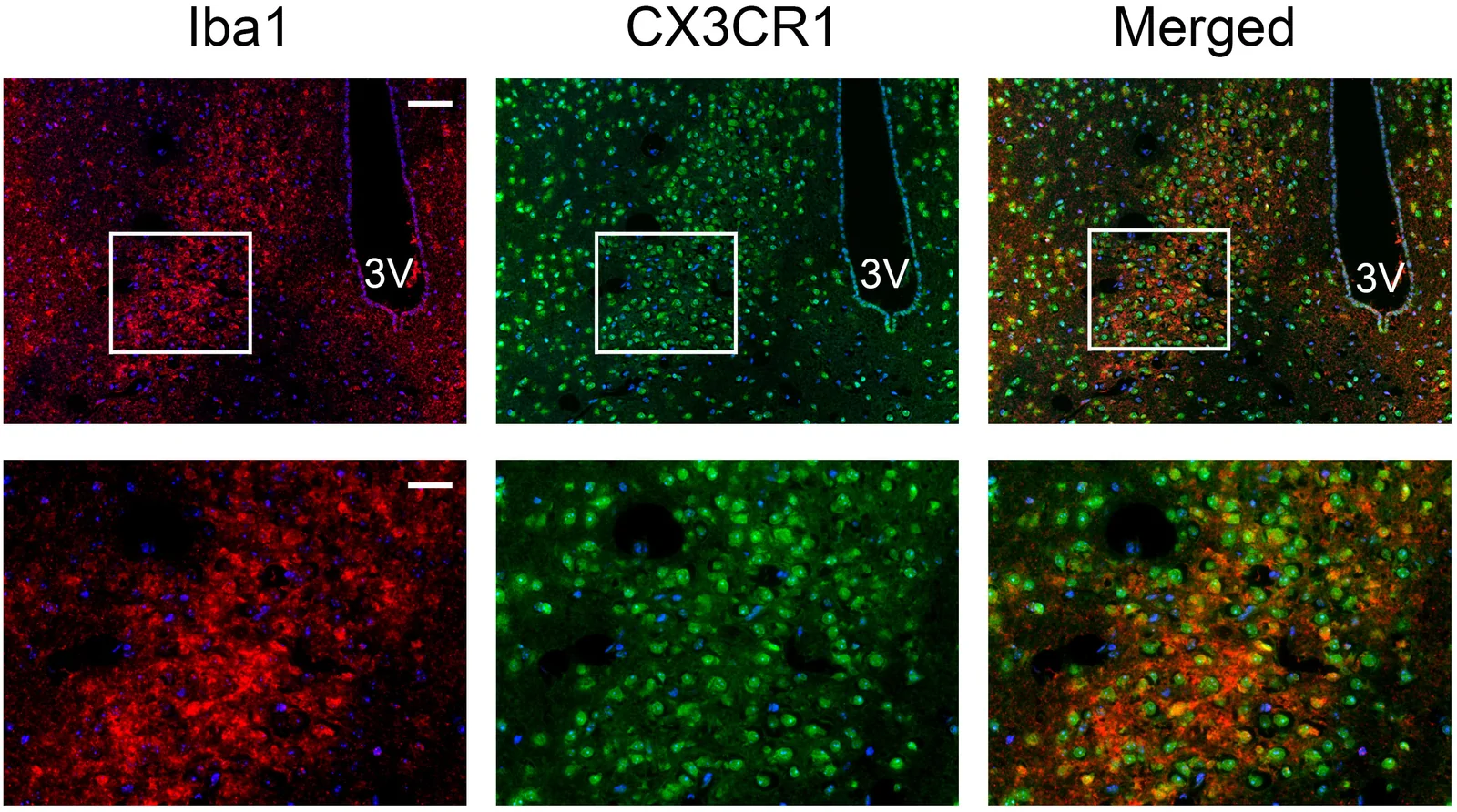
A double-color immunofluorescence analysis of CX3CR1-expressing cells in the paraventricular nuclei (PVN) after RS on WT mice. 3V, the third ventricle. Representative results from 4 animals are shown. Frames in the upper column were displayed at a higher magnification in the lower column. Scale bar in upper column, 50 μm; lower column, 20 μm.

## Discussion

Stress-induced thymic involution is a complex process involving neuroendocrine activation, inflammatory responses, and thymocyte apoptosis (1, 2). In this study, we provide the first evidence that the CX3CL1–CX3CR1 axis plays a critical role in acute stress-induced thymic atrophy. Using *Cx3cr1*-deficient mice, we demonstrated that CX3CR1 signaling promotes thymic involution through macrophage-associated apoptotic mechanisms and regulation of neuroimmune responses.

The HPA axis and glucocorticoid signaling are central regulators of stress-induced thymocyte depletion (2). In line with this, increased endogenous corticosterone has been associated with thymic atrophy under diverse experimental conditions, including Bis gene deletion, ethanol exposure, and protein malnutrition (23, 25, 26). Moreover, blockade of endogenous glucocorticoid signaling attenuated restraint stress-induced thymocyte apoptosis and thymic involution, further supporting a central role for glucocorticoids in this process (2). To directly examine whether exogenous CORT could reproduce the thymic changes observed after RS, we additionally administered CORT to WT and *Cx3cr1^−/−^* mice. A single administration of CORT resulted in a modest tendency toward a reduction in the thymus weight/body weight ratio in both genotypes at 24 h after administration, although the differences compared with vehicle-treated mice did not reach statistical significance (**Supplementary Figure 3**). Furthermore, no significant difference in the response to CORT administration was observed between WT and *Cx3cr1^−/−^* mice. Thus, a single CORT administration did not fully reproduce the significant thymic involution observed after repeated restraint stress. Importantly, these findings do not exclude the established contribution of glucocorticoids to stress-induced thymic involution; rather, they suggest that additional mechanisms may contribute to the pronounced thymic atrophy induced by repeated RS.

A major finding of this study is that restraint stress markedly enhanced CX3CL1 and CX3CR1 expression in thymic F4/80-positive macrophages. CX3CR1 deficiency significantly reduced stress-induced thymic atrophy, preserved cortical architecture, and suppressed thymocyte apoptosis. Moreover, restraint stress increased FasL expression in thymic macrophages, whereas this response was attenuated in *Cx3cr1^-/-^* mice. Together with the reduced caspase-3 activation in cortical thymocytes, these findings support a model in which CX3CR1 signaling promotes a local macrophage–thymocyte apoptotic pathway through FasL, thereby complementing the systemic glucocorticoid-dependent mechanism of stress-induced thymic involution.

In addition to local thymic mechanisms, the expression of CX3CR1 in Iba1-positive microglia raises the possibility that central CX3CL1–CX3CR1 signaling may modulate the neuroendocrine response to restraint stress, thereby influencing peripheral CORT production and subsequent thymic involution. Recent studies have highlighted the involvement of CX3CR1 in microglial responses to environmental and psychological stressors (20, 27). In particular, CX3CL1–CX3CR1 signaling has been shown to modulate microglial activation and functional phenotype under stressful conditions (20, 27). Thus, CX3CL1–CX3CR1-mediated microglial regulation may contribute to systemic neuroendocrine responses during psychological stress. However, further studies are required to determine whether hypothalamic CX3CR1 directly regulates HPA axis activity in our restraint stress model.

This study has some limitations. Although our findings suggest an important contribution of CX3CR1-expressing macrophages, studies using macrophage-specific CX3CR1 deletion or bone marrow chimera models will be required to definitively establish the cellular source responsible for thymic protection. In contrast to the marked protection observed in *Cx3cr1*-deficient mice, systemic administration of anti-CX3CL1 neutralizing antibody failed to attenuate restraint stress-induced thymic atrophy. This discrepancy may be explained by several possibilities. First, CX3CL1 exists as both a soluble chemokine and a membrane-bound adhesion molecule (28). In the present study, CX3CL1 was predominantly detected in F4/80-positive macrophages within the thymus, suggesting that membrane-associated CX3CL1 may act locally through cell–cell interactions. Such spatially restricted signaling may not be efficiently blocked by systemically administered neutralizing antibody. Second, the rapid development of thymic involution after restraint stress may require high and sustained antibody concentrations within the thymic microenvironment, and incomplete tissue penetration or insufficient neutralization may have limited the effect of antibody treatment. Third, genetic *Cx3cr1* deficiency may alter macrophage differentiation, localization, or activation before stress exposure, whereas acute CX3CL1 blockade may not fully reproduce these developmental or cellular effects. Therefore, the negative result of CX3CL1 neutralization does not necessarily exclude involvement of the CX3CL1–CX3CR1 pathway, but it indicates that the present data more directly support a role for CX3CR1-expressing cells in stress-induced thymic involution. Future studies using *Cx3cl1*-deficient mice, conditional *Cx3cr1* deletion, or assessment of antibody penetration and neutralizing activity in thymic tissue will be necessary to determine whether membrane-bound CX3CL1 is indispensable for this process.

In conclusion, our study identifies CX3CR1 signaling as an important contributor to restraint stress-induced thymic involution. Our findings suggest that CX3CR1 contributes to thymocyte apoptosis through mechanisms involving macrophage-associated FasL expression, while also modulating the corticosterone response to restraint stress. These findings support a model in which local immune mechanisms and systemic neuroendocrine responses may cooperatively contribute to stress-induced thymic involution and reveal a previously unrecognized role for CX3CR1 signaling in the regulation of thymic responses to repeated stress.

## Data Availability

The original contributions presented in the study are included in the article/**Supplementary Material**. Further inquiries can be directed to the corresponding author.

## Supplementary material

Supplementary Figure 1 The expression of chemokines, chemokine receptors, and inflammatory cytokines in the thymus of WT and *Cx3cr1*^-/-^ mice before and after RS. Real-time Reverse Transcription Polymerase Chain Reaction (RT-PCR) analysis on the gene expression of *Ccl2, Ccl3, Ccl4, Ccl5, Ccr2, Ccr5, Il1b, Il6, Il12p35* and *Il12p40*. All values represent mean ± SEM (n = 4 mice).

Supplementary Figure 2 Effect of anti-CX3CL1 on RS-induced thymic atrophy. WT mice were injected with CX3CL1 neutralizing antibody or control IgG, and RS-induced thymic atrophy was analyzed (n = 4 mice).

Supplementary Figure 3 Effect of exogenous corticosterone administration on thymic weight in WT and *Cx3cr1*^−/−^ mice. WT and *Cx3cr1*^−/−^ mice received a single intraperitoneal injection of corticosterone (CORT; 4 mg/mouse in 100 μL olive oil) or vehicle (100 μL olive oil). Twenty-four hours after administration, mice were euthanized, and the thymi were collected and weighed. (a) Representative macroscopic appearance of the thymi. (b) Thymus weight/body weight ratio (mg/g). Data are expressed as mean ± SEM (n = 4 mice per group). No statistically significant differences were observed among the groups. Statistical analysis was performed using two-way ANOVA followed by *post hoc* test.
