# Supplementary material for: CX3CL1–CX3CR1 axis promotes restraint stress-induced thymic involution through macrophage-mediated thymocyte apoptosis
Source: Front Immunol. 2026 Sep 15;17:1930342. doi: 10.3389/fimmu.2026.1930342 (PMC13619386; doi:10.3389/fimmu.2026.1930342)
Supplement: Supplementary file 1 [file DataSheet1.pdf]

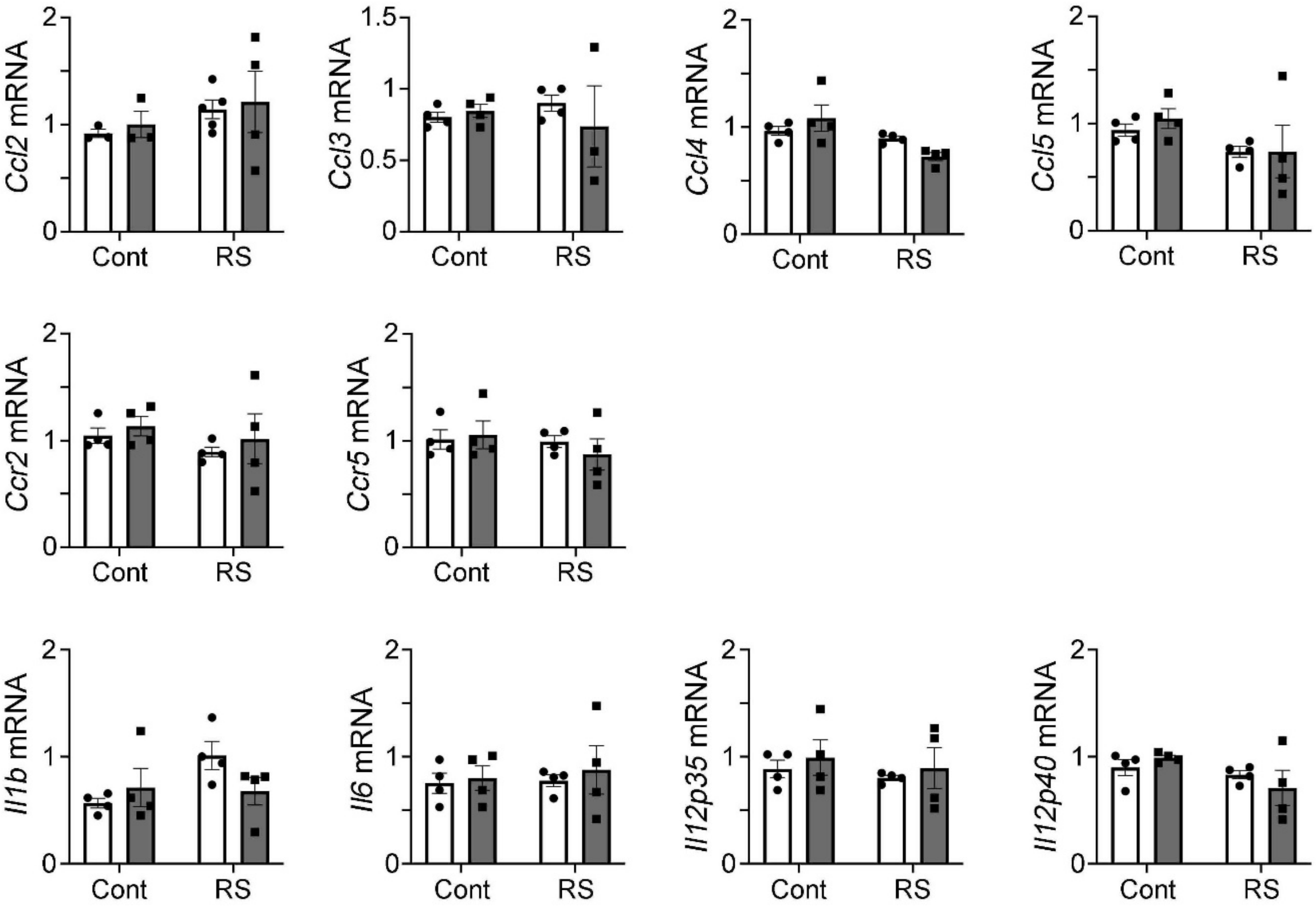

Figure S1. The expression of chemokines, chemokine receptors, and inflammatory cytokines in the thymus of WT and *Cx3cr1*$^{-/-}$ mice before and after RS. Real-time Reverse Transcription Polymerase Chain Reaction (RT-PCR) analysis on the gene expression of *Ccl2, Ccl3, Ccl4, Ccl5, Ccr2, Ccr5, Il1b, Il6, Il12p35* and *Il12p40*. All values represent mean ± SEM (n = 4 mice).

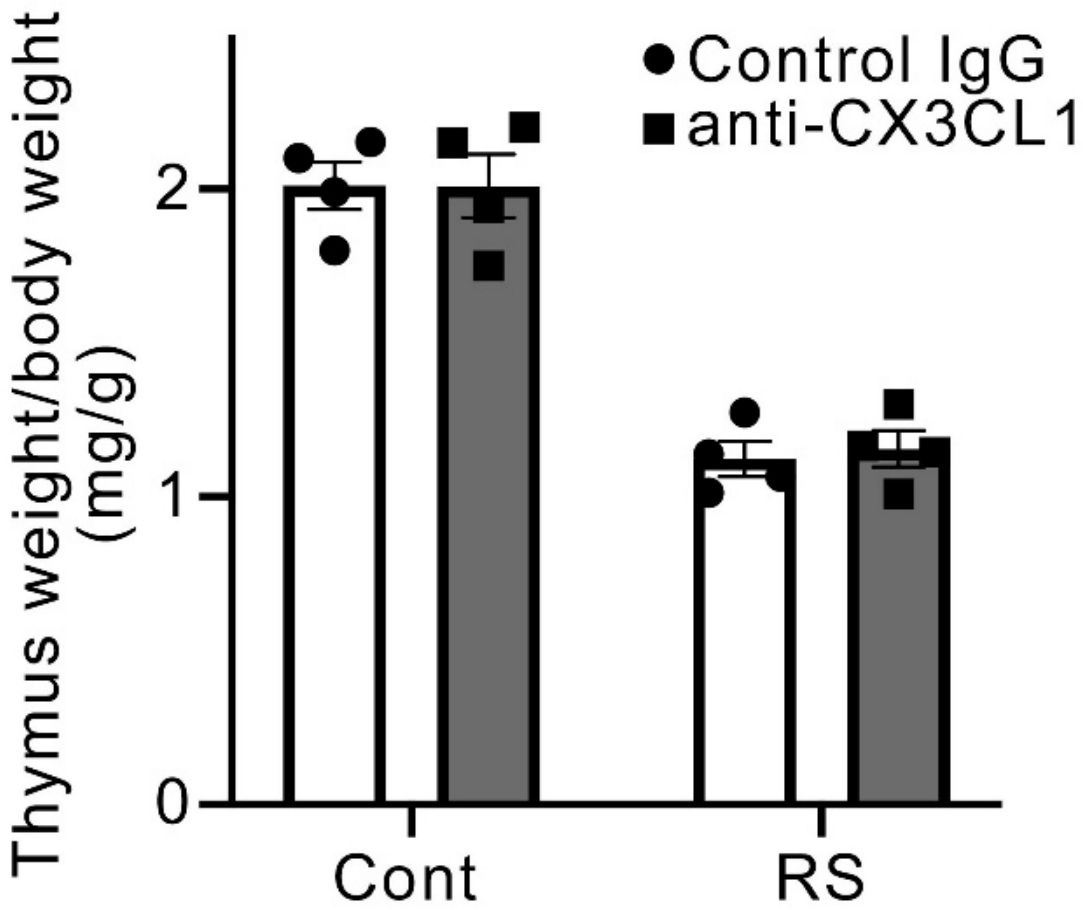

Figure S2. Effect of anti-CX3CL1 on RS-induced thymic atrophy. WT mice were injected with CX3CL1 neutralizing antibody or control IgG, and RS-induced thymic atrophy was analyzed (n = 4 mice).

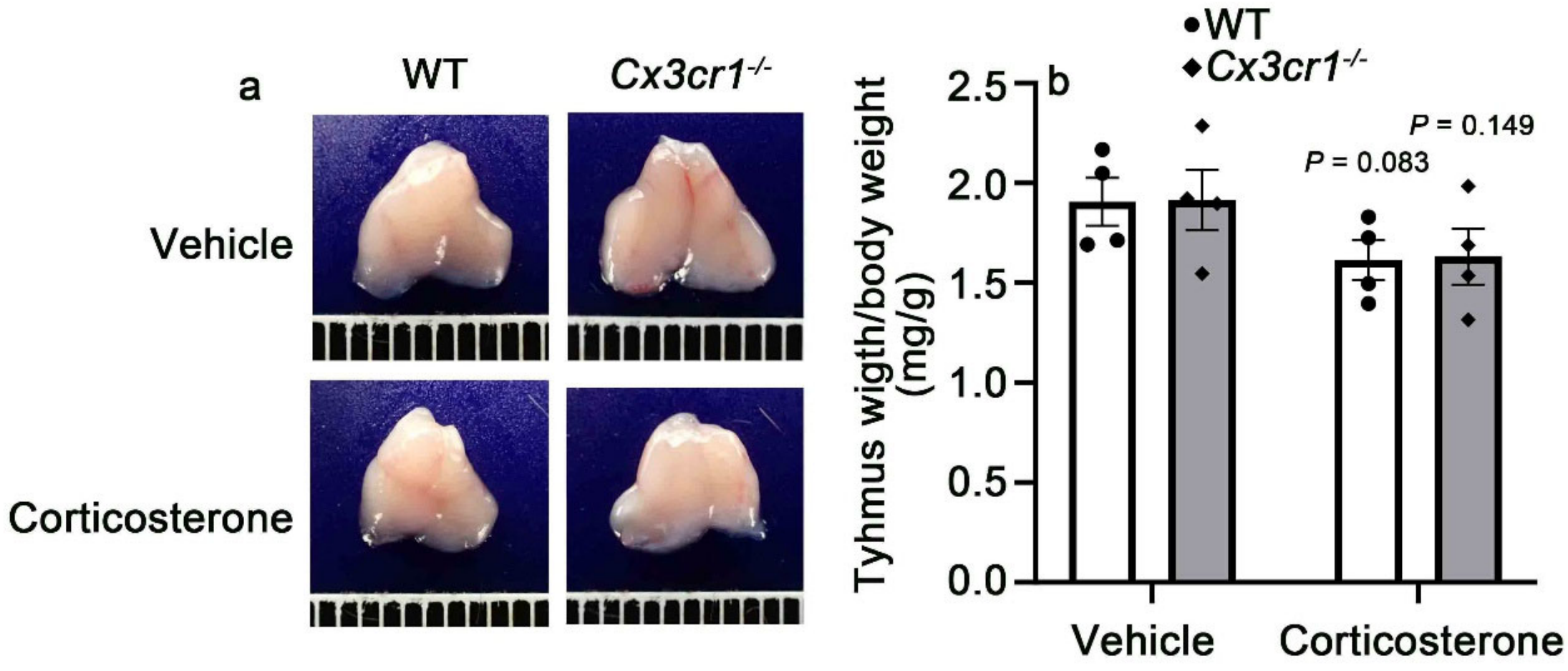

Figure S3. Effect of exogenous corticosterone administration on thymic weight in WT and *Cx3cr1*$^{-/-}$ mice. WT and *Cx3cr1*$^{-/-}$ mice received a single intraperitoneal injection of corticosterone (CORT; 4 mg/mouse in 100 µL olive oil) or vehicle (100 µL olive oil). Twenty-four hours after administration, mice were euthanized, and the thymi were collected and weighed. (a) Representative macroscopic appearance of the thymi. (b) Thymus weight/body weight ratio (mg/g). Data are expressed as mean ± SEM (n = 4 mice per group). No statistically significant differences were observed among the groups. Statistical analysis was performed using two-way ANOVA followed by post hoc test.
